# Supplementary material for: Unraveling the role of salt-sensitivity genes in obesity with integrated network biology and co-expression analysis
Source: PLoS One. 2020 Feb 6;15(2):e0228400. doi: 10.1371/journal.pone.0228400 (PMC7004317; doi:10.1371/journal.pone.0228400)
Supplement: S2 Table — (PDF) [file pone.0228400.s002.pdf]

S2 Table: The list of Salt Sensitive Genes analyzed in the present study

Gene	Name
ACE	angiotensin I converting enzyme
ACE2	angiotensin I converting enzyme 2
ADD1	adducin 1
ADRB2	adrenoceptor beta 2
AGT	angiotensinogen
AGTR1	angiotensin II receptor type 1
AGTR2	angiotensin II receptor type 2
ANPEP	alanyl aminopeptidase, membrane
ATP6AP2	ATPase H+ transporting accessory protein 2
CLCNKB	chloride voltage-gated channel Kb
CMA1	chymase 1
CPA3	carboxypeptidase A3
CTSA	cathepsin A
CTSG	cathepsin G
CYP11B1	cytochrome P450 family 11 subfamily B member 1
CYP11B2	cytochrome P450 family 11 subfamily B member 2
CYP17A1	cytochrome P450 family 17 subfamily A member 1
CYP3A5	cytochrome P450 family 3 subfamily A member 5
ENPEP	glutamyl aminopeptidase
GNAI2	G protein subunit alpha i2
GNB3	G protein subunit beta 3
GRK4	G protein-coupled receptor kinase 4
HSD11B2	hydroxysteroid 11-beta dehydrogenase 2
KCNJ1	potassium voltage-gated channel subfamily J member 1
KLK1	kallikrein 1
KLK2	kallikrein related peptidase 2
LNPEP	leucyl and cystinyl aminopeptidase
MAS1	MAS1 proto-oncogene, G protein-coupled receptor
MME	membrane metalloendopeptidase
SLC24A3	solute carrier family 24 member 3
SLC8A1	solute carrier family 8 member A1
NEDD4L	neural precursor cell expressed, developmentally down-regulated 4-like, E3 ubiquitin protein ligase
NLN	neurolysin
PRCP	prolylcarboxypeptidase
PREP	prolyl endopeptidase
PRKG1	protein kinase cGMP-dependent 1
REN	renin
SCNN1A	sodium channel epithelial 1 alpha subunit

SCNN1B	sodium channel epithelial 1 beta subunit
SCNN1D	sodium channel epithelial 1 delta subunit
SCNN1G	sodium channel epithelial 1 gamma subunit
SGK1	serum/glucocorticoid regulated kinase 1
SLC4A4	solute carrier family 4 member 4
SLC4A5	solute carrier family 4 member 5
TH	tyrosine hydroxylase
THOP1	thimet oligopeptidase 1
WNK1	WNK lysine deficient protein kinase 1
