# Supplementary material for: Unraveling the role of salt-sensitivity genes in obesity with integrated network biology and co-expression analysis
Source: PLoS One. 2020 Feb 6;15(2):e0228400. doi: 10.1371/journal.pone.0228400 (PMC7004317; doi:10.1371/journal.pone.0228400)
Supplement: Attachment — Submitted filename: reviewer_comments.docx [file pone.0228400.s004.docx]

The manuscript describes a study by the authors which identifies network of salt sensitivity genes and their co-expressed genes to trace the molecular pathways connected to obesity in visceral adipose tissues using gene expression and weighted protein interaction network. This work systematically outlines an integrated bioinformatics pipeline for figuring out the most indispensable key signatures from the interactome Salt Sensitivity Protein Interaction Network. Most recent bioinformatics tools and software have been employed in this investigation, hence it proves the gene's role or its association to Obesity. Touching upon a burning problem like obesity is a good analysis. The manuscript is interesting and publishable in PLOS one journal after the minor issues are addressed by authors.

1. In the introduction section, the authors have mentioned “We have also adopted another novel concept i.e. gene-gene correlation…….” replace “novel concept” with some other suitable word as the mentioned concept is not novel
2. References needed for “The statistical difference between differentially expressed genes (DEGs) was computed using unpaired t-test measure among healthy and obese samples.”
3. References needed for protein interaction databases mentioned in the section the *section 2.3*
4. References needed for “Next, the plugin NetworkAnalyzer, calculated the degree centrality betweenness….” Mentioned in the section 3.2
5. In the section “3.5 Co-expression analysis” Eight among the 23 co-expressed genes were not previously reported for the disease obesity via gene ontology analysis. Replace the phrase ‘gene ontology’ with suitable word like functional enrichment or gene set enrichment analysis
6. In the section, 4 Discussion,

“Local parameter DC and global parameter BC were used to dissect the complex interactome.”

The terms Local parameter and global parameter are not given any explanation in method or results section
